# Knee Wounds: A Novel Approach to Reconstruction

**Published:** 2013-02-28

**Authors:** Portia Thurmond, Michael M. Van Vliet, Emily B. Ridgway

**Affiliations:** Department of Plastic and Reconstructive Surgery, Dartmouth-Hitchcock Medical Center, Lebanon, NH

**Figure:**
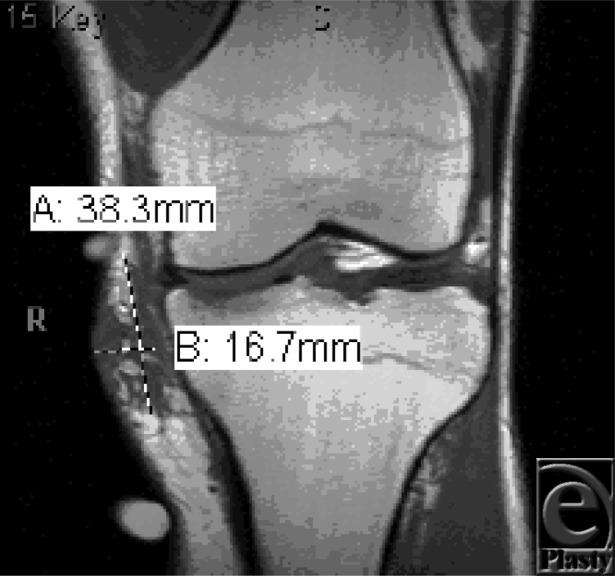


## DESCRIPTION

A 58-year-old man without medical comorbidities presented with a medial knee region mass. Tissue biopsy revealed a sarcoma and the patient underwent resection. Subsequent soft tissue reconstruction followed by radiotherapy was necessary.

## QUESTIONS

1. **What is the current recommendation for coverage of knee wounds?**
2. **What are the complications associated with local muscle coverage?**
3. **What other options are there besides local muscle?**
4. **Describe the advantages and specifics of the medial sural artery perforator flap.**

## DISCUSSION

The pedicled gastrocnemius flap is the preferred time-honored approach for soft tissue coverage of knee wounds following trauma or tumor extirpation. The flap can be raised by harvesting either the medial or lateral portions of the muscle. The medial and lateral gastrocnemius flaps have independent blood supplies, the medial and lateral sural arteries, respectively. Both vessels originate from the popliteal artery and usually measure greater than 1 mm in diameter. The medial flap is more commonly used given the larger size of the medial portion of the muscle. While these flaps can be harvested as free flaps, they are more commonly used for local tissue coverage.^1^^-^2

Limitations of this flap include the morbidities of functional impairment directly associated with loss of muscle and longer operative and recovery times. In addition, and arguably most importantly, the donor site can be unsightly causing significant contour deformity. Trauma patients requiring gastrocnemius coverage of knee wounds are often young making the contour deformity even more unacceptable. The contour depression resulting from loss of muscle has led surgeons to seek alternative therapy.^1^^-^2

**Figure 1:**
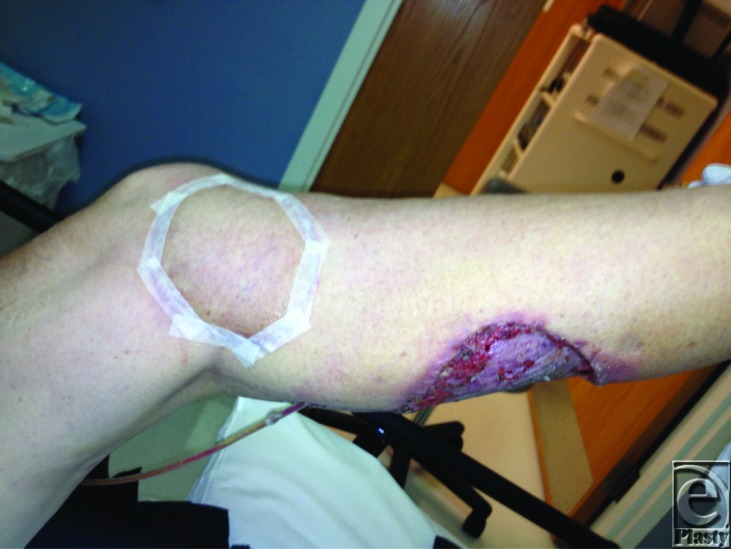
Medial sural artery perforator flap in place at 1 week after surgery.

With this in mind, we consider the medial sural artery perforator flap to serve as an alternative option in proximal lower leg and knee wound reconstruction. Figure 1 shows the flap in place 1 week after surgery with split thickness skin graft (STSG) to the donor site. Perforators of the sural artery have been the basis for free and pedicled fasciocutaneous flaps applied in a variety of clinical settings in the past.^3^^-^7 In 2001, Cavadas et al^3^ first described the anatomy and use of the flap as a fasciocutaneous free flap. Since then, both Kim et al^5^ and Shim et al^6^ have shared case series in which the flap is used as a pedicled flap with varied pedicle length and flap size.

Cadaver studies show that medial sural artery has an average of 2 perforators (range 1-5) and is accompanied by 2 venae comitantes. The first perforator is located at an average distance of 10.2 ± 0.02 cm from the popliteal artery while the second perforator is located at an average distance of 15.9 cm. The perforators can be easily dissected through the head of the medial gastrocnemius muscle. The average diameter of the larger and more proximal perforator is 0.9 mm, while the smaller and more distal perforator has an average diameter of 0.5 mm. It is estimated that the possible size of the flap that can be raised from superficial to the medial head of the gastrocnemius muscle is 8.2 cm × 13.3 cm. The flap salvages the main pedicle, limits functional impairment, and minimizes donor site morbidity.^8^

In summary, proximal calf and knee wounds present a reconstructive challenge. Often these patients are young, active patients with wounds resulting from trauma and sacrifice of a muscle is not desirable. The medial sural perforator island flap has been shown to have reliable perforator locations and a reliable blood supply without sacrifice of the distal foot blood supply or the underlying muscle. There is minimal functional deficit of donor site and good esthetic result of the defect. We hope this will be increasingly used for reconstruction of the upper one third of lower leg and knee.

## Figures and Tables

**Figure 2:**
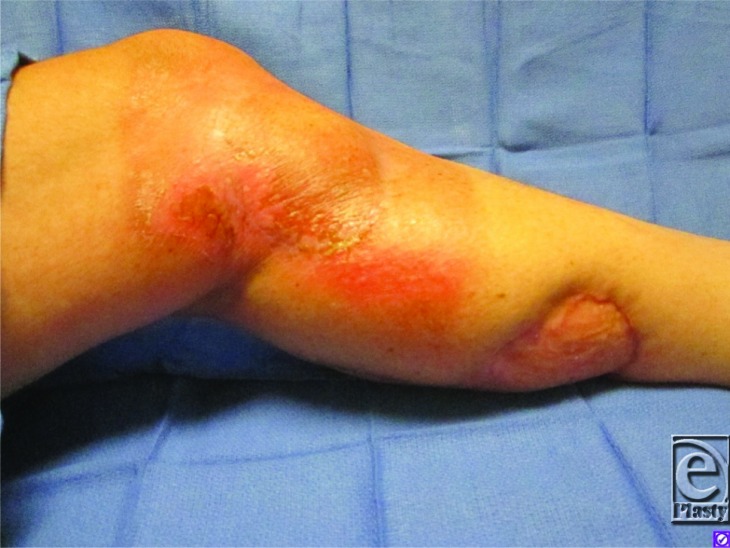
Healed flap with excellent viability at 4 months after surgery.
